# Identification of stem cell-related subtypes and risk scoring for gastric cancer based on stem genomic profiling

**DOI:** 10.1186/s13287-021-02633-x

**Published:** 2021-10-30

**Authors:** Renshen Xiang, Wei Song, Jun Ren, Jing Wu, Jincheng Fu, Tao Fu

**Affiliations:** 1 Department of Gastrointestinal Surgery II, Renmin Hospital of Wuhan University, Wuhan, 430060 Hubei Province China; 2 Central Laboratory, Renmin Hospital of Wuhan University, Wuhan, 430060 Hubei Province China

**Keywords:** Gastric cancer, Stem cell gene sets, Immune infiltration, Tumor mutation burden, Therapeutic response, Risk score

## Abstract

**Background:**

Although numerous studies demonstrate the role of cancer stem cells in occurrence, recurrence, and distant metastases in gastric cancer (GC), little is known about the evolving genetic and epigenetic changes in the stem and progenitor cells. The purpose of this study was to identify the stem cell subtypes in GC and examine their clinical relevance.

**Methods:**

Two publicly available datasets were used to identify GC stem cell subtypes, and consensus clustering was performed by unsupervised machine learning methods. The cancer stem cell (CSC) typing-related risk scoring (RS) model was established through multivariate Cox regression analysis.

**Results:**

Cross-platform dataset-based two stable GC stem cell subtypes, namely low stem cell enrichment (SCE_L) and high stem cell enrichment (SCE_H), were prudently identified. Gene set enrichment analysis revealed that the classical oncogenic pathways, immune-related pathways, and regulation of stem cell division were active in SCE_H; ferroptosis, NK cell activation, and post-mutation repair pathways were active in SCE_L. GC stem cell subtypes could accurately predict clinical outcomes in patients, tumor microenvironment cell-infiltration characteristics, somatic mutation landscape, and potential responses to immunotherapy, targeted therapy, and chemotherapy. Additionally, a CSC typing-related RS model was established; it was strongly independent and could accurately predict the patient’s overall survival.

**Conclusions:**

This study demonstrated the complex oncogenic mechanisms underlying GC. The findings provide a basis and reference for the diagnosis and treatment of GC.

**Supplementary Information:**

The online version contains supplementary material available at 10.1186/s13287-021-02633-x.

## Introduction

Globally, gastric cancer (GC) is one of the most common gastrointestinal malignancies with high incidence and mortality rates. According to the latest data from the International Cancer Research Agency, annually, there are approximately 930,000 new cases and 700,000 deaths, and GC ranks fourth and second in morbidity and mortality, respectively, among all malignant tumors [1]. To date, despite the emergence of new diagnostic and therapeutic methods, the postoperative prognoses of some patients remain poor, and it is mainly attributed to the recurrence and metastases in GC [2]. Thus, it is necessary to investigate in detail at the molecular level, to overcome these shortcomings.

As early as 1959, Makino et al. [3] hypothesized that tumor cells may originate from cancer stem cells (CSCs). By 1997, Dick et al. [4] were able to isolate human acute myeloid leukemia stem cells, which for the first time proved the existence of CSCs. Currently, CSCs have been successfully identified in several solid tumors, and a growing body of evidence shows that CSCs which arise from epigenetic mutations in the stem or progenitor cells account for 0.1% of tumor cells [5–7]. CSCs are usually dormant in cancer nests as the DNA replication is inactive, and thus, they can avoid DNA damage induced by chemotherapy drugs [6, 7]. Moreover, CSCs have strong repair ability after DNA damage and can maintain stable genetic inheritance [6, 7] regulated by complicated gene regulatory networks in the tumor microenvironment (TME) and tumor cells; these include the Hippo signaling [8], Hedgehog signaling [9], WNT/β-catenin, [10] and NK-kB signaling pathways [11]. CSCs have the ability of infinite proliferation, self-renewal, and multi-directional differentiation [7, 12]. Among these, multi-directional differentiation leads to tumor heterogeneity, which in turn has important impacts on tumor recurrence, metastasis, and drug resistance [13, 14]. In addition, CSCs can secrete immunosuppressive cytokines, such as TGF-β, IL-6, IL-10, and IL-13, which can mediate the immune escape of tumor cells [15, 16].

Currently, there are several studies on molecular typing of GC, including Lei typing [17], The Cancer Genome Atlas (TCGA) typing [18], and The Asian Cancer Research Group (ACRG) typing [19]. These have elucidated the GC pathogenic mechanisms at the molecular level; however, the origin of tumor heterogeneity has not been demonstrated. Recent evidence suggests that CSCs can act as tumor seeds, and thus may be a potential driving force for heterogeneity [20, 21]. Therefore, the identification of stem cell subtypes could fundamentally indicate the heterogeneity of tumors. In this study, based on the 26 human stem cell gene sets, we classified GC into low stem cell enrichment (SCE_L) and high stem cell enrichment (SCE_H) types. We further verified the stability and credibility of this classification method using cross-platform datasets and various algorithms. More importantly, this study thoroughly discussed the biological pathways, TME status, immune cell infiltration, immune checkpoint gene (ICG) expression, somatic mutation landscape, and potential sensitivity to targeted therapy and chemotherapy between the two stem cell subtypes. Various genetic and epigenetic changes in the evolution of stem cells were systematically examined, and these findings may provide new insights for the clinical diagnoses, treatments, and prognostic judgments for GC.

## Materials and methods

### Acquisition and processing of publicly available data

In this study, the patient transcriptional profiles and corresponding clinicopathological data, including age, sex, tumor grade, TNM stage, survival time, and survival status, were obtained from the TCGA (http://cancergenome.nih.gov/) and Gene Expression Omnibus databases (GSE84437, https://www.ncbi.nlm.nih.gov/geo/). A total of 32 normal and 808 (375 from TCGA and 433 from GSE84437) GC samples were acquired from the two publicly available open data sources. After processing the original data using the Perl software, GC samples with survival time < 30 days, ambiguous survival status, and unclear clinicopathological characteristics were excluded. In addition, somatic mutation data of GC patients were obtained from the TCGA database and 26 human stem cell gene sets were collected from the StemChecker portal (http://StemChecker.sysbiolab.eu/) [22] and previous literature [23].

### Identification of GC stem cell subtypes based on stem genomic profiling

Single-sample gene set enrichment analysis (ssGSEA) was performed using the ‘GSVA’ package the enrichment of each GC sample in the 26 stem cell gene sets was quantified. The ‘ConsensusClusterPlus’ package was used for consensus clustering and identification of GC stem cell subtypes. K-means algorithm and cumulative distribution function (CDF) curve were used to determine the best number of subtypes, and 50 iterations with maxK = 9 were performed for stable subtypes. The stability of GC stem cell subtypes was verified by principal component analysis (PCA) and t-distributed stochastic neighbor embedding (tSNE) algorithms. Kaplan–Meier curves were used to evaluate the survival differences among GC stem cell subtypes. The ‘ggplot2’ package was used to visualize the proportion of existing GC subtypes in each stem cell subtype.

### Differential expression and functional analyses of GC stem cell subtypes

Based on the set criteria of |log2[fold change (FC)]|> 0.5 and a false discovery rate (FDR) < 0.05, we investigated the differentially expressed genes (DEGs) among GC stem cell subtypes in TCGA and GSE84437 cohorts. Subsequently, the biological processes (BP) were analyzed for the DEGs, and gene set enrichment analysis (GSEA) with log2(FC) as the phenotype was performed using the ‘Clusterprofiler’ package.

### TME scores, immune cell fractions, and ICG expression for GC stem cell subtypes

TME scores (immune/stromal scores and tumor purity) for each sample were calculated using the ‘ESTIMATE’ package. The fraction of 22 immune cells in each sample was identified by using the CIBERSORT algorithm. In addition, 26 ICG representatives were obtained through extensive literature review [24–28], and the levels of ICGs in GC stem cell subtypes were investigated by differential expression analysis.

### Somatic mutation landscape and tumor mutation burden (TMB) of GC stem cell subtypes based on the TCGA cohort

The top 30 genes (ranked by somatic mutation frequency) of GC stem cell subtypes were visualized using the ‘maftools’ package. Using the annotation file, the TMB of each sample was calculated through the Perl software. Kaplan–Meier analysis along with log-rank test was used to examine the prognostic value of TMB in GC, and observe the co-survival results for TMB and GC stem cell subtypes.

### Prediction of targeted therapeutic and chemotherapeutic responses for GC stem cell subtypes

A total of 6 genes for GC targeted therapy were obtained through extensive literature review [29–33]. We investigated their levels in GC stem cell subtypes based on differential expression analysis. After log2-scale transformation of RNA-seq expression profiles, we used the “pRRophetic” package in R and estimated the half-maximum inhibitory concentration, IC50, using ridge regression, to predict the chemotherapeutic response of each sample in the TCGA and GSE84437 datasets based on the Genomics of Drug Sensitivity in Cancer database (GDSC, https://www.cancerrxgene.org/); twelve chemotherapeutic agents were selected, including camptothecin, methotrexate, mitomycin C, doxorubicin, gemcitabine, paclitaxel, imatinib, bleomycin, docetaxel, sunitinib, cisplatin, and vinblastine. Based on the GDSC training set, tenfold cross-validation was used to evaluate the accuracy of the prediction.

### Potential small molecule drugs based on the connectivity map database

The overlapping DEGs between the TCGA cohort and the GSE84437 dataset were incorporated into the connectivity map (CMAP) database to mine for small-molecule drugs that could be used in therapy or drug research [34]. Briefly, first, the gene names of DEGs were converted to probe ID. Second, we registered an account on the CMAP portal. Third, using the “Quick Query” option in the CMAP database, we uploaded the files containing the probe IDs and finally obtained the potential small molecule drugs.

### Development and validation of prognostic risk scoring model

After log2-scale transformation of DEG expressions for GC stem cell subtypes, univariate analyses were performed for GSE84437 and TCGA cohorts, and the co-prognostic genes were used for further analysis. Subsequently, the GSE84437 dataset was considered as the training set and the TCGA cohort as the validation set to construct a risk scoring (RS) model for predicting the overall survival (OS) of patients through multivariate Cox regression analysis. The mean value of the RS model was used to divide the patients into high- and low-risk groups. The RS was calculated as the sum of the products of gene expression levels and their coefficients as follows:

$${\text{RS}}=\sum_i^k ({\text{Exp}}_i \times{\text{Coe}}_i)$$

where ‘*i*’ and ‘*k*’ represent the *i*th gene and the total number of genes, respectively. Kaplan–Meier analysis and receiver operating characteristic (ROC) curves were used to evaluate the accuracy and prediction efficiency. PCA and tSNE algorithms were used to evaluate the stability of the RS model. Univariate analysis was used for calculating the hazard ratios (HR) of the factors, and further, multivariate analysis was used to determine the independent prognostic factors. The results were visualized using the ‘forestplot’ package.

Difference analysis was used to compare the RS between SCE_H and SCE_L groups. The ‘h.all.v7.4.symbols’, containing multiple well-defined biological signatures, were downloaded from the MSigDB database (https://www.gsea-msigdb.org/gsea/msigdb/index.jsp). The enrichment of each well-defined biological signature was quantified using the ssGSEA algorithm. Differential analysis was performed to determine the activity of each well-defined biological signature between SCE_H and SCE_L types. The connection between RS and TME cell infiltration was analyzed using Spearman’s correlation analysis.

### Detection of the relative expression levels of genes in the RS model

A total of 14 pairs of GC samples (Additional file 1: Table S1) were collected and the relative expression of genes in the RS model was detected. The study was approved by the Ethics Committee of Renmin Hospital of Wuhan University (No. NCT03972956V1.1), and we have obtained informed consents from the patients for using GC samples (No. SAMPGICU2019-2). Total RNA was extracted using Tissue Total RNA Isolation Kit (Foregene, Wuhan, China) as per the manufacturer’s protocol and RNA (2 μg) was reverse transcribed to cDNA using the PrimeScriptTM RT reagent Kit (TaKaRa, Osaka, Japan). Quantitative PCR (qPCR) was performed using the SYBR Green qPCR Supermixes (Bio-Rad) on the CFX 192 Connect Real-Time PCR system (Bio-Rad, USA). The qPCR analysis was performed in triplicates with the designed primers (Table 1). The relative expression was normalized to GAPDH using the 2-ΔΔCT method.

**Table 1:** List of primers

Gene	Primer sequence (5′–3′)
SLIT2	Forward: GCACCATTGAAAGAGGAGCA
	Reverse: GCTTTCCTTGGGATTGCCTG
SFRP2	Forward: ACCGAGGAAGCTCCAAAGGTA
	Reverse: GAGCCACAGCACCGATTTCT
SCRG1	Forward: CATTTCTGGGATGGGAAGGGA
	Reverse: GTGGGAAATCAGGAATGGTGTT
MFAP5	Forward: CAGCGTAAGAGGAGAGAGACAC
	Reverse: CAGCAAGAAACAGCAGCACCT
EFEMP1	Forward: TGAAATGCAGACTGGCCGAA
	Reverse: TCTACAGTTGTGCGTCCCTG
COL8A1	Forward: AAGGAGATGCCCCACTTGC
	Reverse: GGACCTTGTTCCCCTCGTAA
ABCA8	Forward: AAGAACGCAAAACAGACCGC
	Reverse: TTTGGCATCAGGGATGTGCT

### Statistical analysis

For the quantitative data, the normally distributed variables were analyzed using a Student’s t-test, whereas the non-normally distributed variables were estimated using the Wilcoxon rank–sum test. For comparisons of more than two groups, the one-way analysis of variance and Kruskal–Wallis tests were used as the parametric and nonparametric methods, respectively. Kaplan–Meier statistics and log-rank tests were used for survival analysis. All statistical analyses were performed in R and Perl, and *P* < 0.05 was considered statistically significant.

## Results

### Identification and validation of GC stem cell subtypes based on stem cell gene sets

**Fig. 1:**
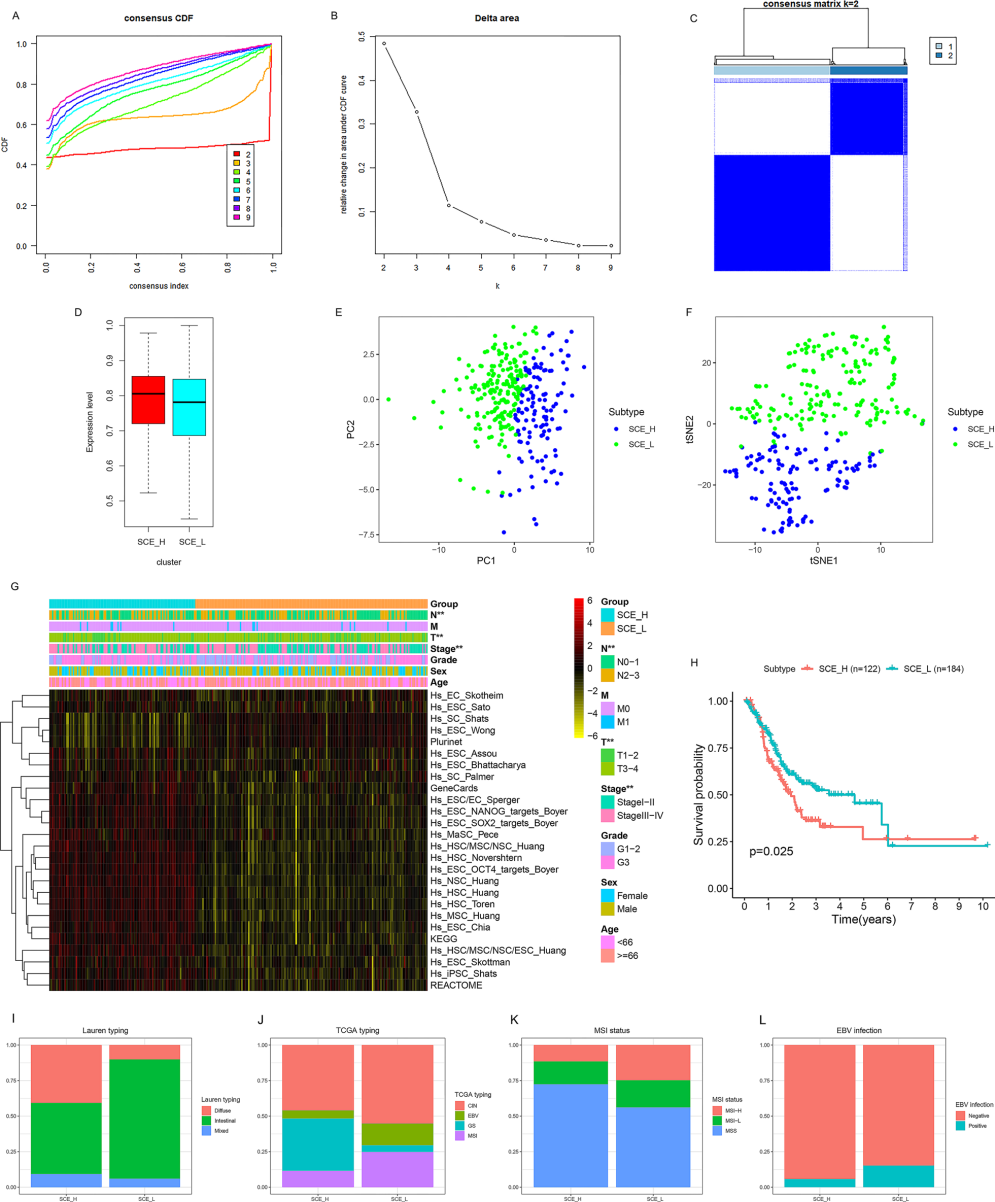
Identification of GC stem cell subtypes based on the TCGA database. **a–c** Two stable stem cell subtypes were identified using consensus clustering analysis according to the K-means algorithm and CDF curve. **d** GC subtypes were classified as SCE_L and SCE_H based on 26 stem cell gene sets. Clustering of patients belonging to SCE_L and SCE_H in the TCGA cohort based on **e** PCA and **f** tSNE algorithm. **g** The expression of 26 stem cell gene sets and the proportion of clinicopathological features in SCE_L and SCE_H. **h** Kaplan–Meier analysis of GC stem cell subtypes. **i–l** The proportion of **i** Lauren subtypes, **j** TCGA subtypes, **k** MSI subtypes, and **l** EBV infection subtypes in GC stem cell subtypes. Statistical significance: *P < 0.05; **P < 0.01; ***P < 0.001

We quantified the scores of 26 human stem cell gene sets for each GC sample using ssGSEA. Using, consensus clustering analysis, two stable stem cell subtypes according to the K-means algorithm and CDF curves were identified (Fig. 1a–c). Based on the enrichment of the 26 stem cell gene sets in the two subtypes (Fig. 1d, g), SCE_L and SCE_H groups were defined. The PCA and tSNE algorithm demonstrated the strong stability of the two stem cell subtypes (Fig. 1e, f). The T stage (*P* < 0.01), N stage (*P* < 0.01), and TNM stage (*P* < 0.01) were significantly different between the two stem cell subtypes (Fig. 1g), and the pathological results for SCE_H were worse. Kaplan–Meier analysis showed that the OS of SCE_L was significantly better than SCE_H (Fig. 1h, P = 0.025). The results were also verified in the GSE84437 dataset (Additional file 2: Fig. S1).

Furthermore, the relationship between GC stem cell subtypes and Lauren typing (diffuse, intestinal, and mixed), TCGA typing (CIN, EBV, GS, and MSI), microsatellite instability (MSI) status (MSI-H, MSI-L, and MSS), and Epstein-Barr virus (EBV) infection was investigated. We found that SCE_H was characterized by diffuse histological type, GS subtype, MSS, and negative for EBV infection; while SCE_L was characterized by intestinal histological type, MSI, and EBV subtypes, MSI-H, and positive for EBV infection (Fig. 1i–l).

### BP analysis and GSEA based on the TCGA and GSE84437 cohorts

**Fig. 2:**
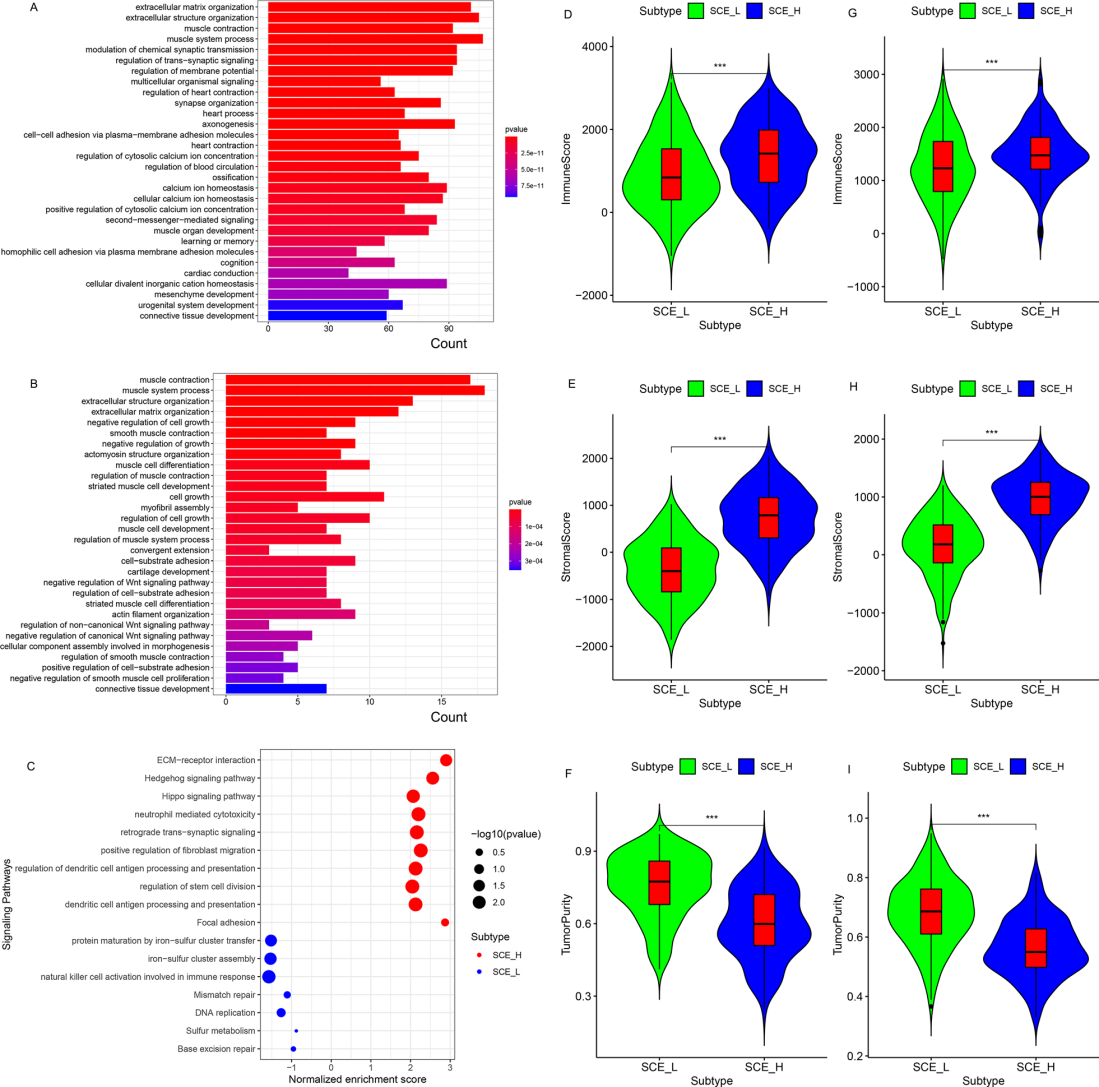
Functional analysis of DEGs (SCE_H/SCE_L) and tumor microenvironment scores for GC stem cell subtypes. BPs based on the GO annotation in the **a** TCGA and **b** GSE84437 cohorts. **c** GSEA based on the common DEGs between the TCGA and GSE84437 cohorts. Comparison of the immune score, stromal score, and tumor purity between SCE_L and SCE_H in the **d**–**f** TCGA and **g–i** GSE84437 cohorts. Statistical significance: *P < 0.05; **P < 0.01; ***P < 0.001

To examine the functional differences between the GC stem cell subtypes, differential expression analysis (SCE_H/SCE_L) was performed and numerous DEGs for gene ontology annotation were accessed. Interestingly, both in the TCGA (Fig. 2a) and GSE84437 datasets (Fig. 2b), BPs for DEGs were associated with TME (e.g. extracellular matrix organization, extracellular structure organization, and positive regulation of cell-substrate adhesion), signal transduction (e.g. modulation of chemical synaptic transmission, regulation of postsynaptic membrane potential and regulation of cation channel activity), tumorigenic pathways (e.g. negative regulation of Wnt signaling pathway), and immune-related pathways (e.g. regulation of neutrophil chemotaxis and regulation of granulocyte chemotaxis). Moreover, GSEA showed that the classical oncogenic pathways (e.g. ECM-receptor interaction, Hedgehog signaling pathway, and Hippo signaling pathway), immune-related pathways (e.g. neutrophil-mediated cytotoxicity and dendritic cell antigen processing and presentation), and regulation of stem cell division were active in SCE_H (Fig. 2c); while ferroptosis (e.g. protein maturation by iron-sulfur cluster transfer and iron-sulfur cluster assembly), NK cell activation (e.g. natural killer cell activation involved in immune response), and post-mutation repair pathways (e.g. mismatch repair, DNA replication, and base excision repair) were active in SCE_L (Fig. 2c). These results lay the molecular foundation for the differential prognoses of GC stem cell subtypes.

### Comparison of TME scores, immune cell fraction, and ICG expression between GC stem cell subtypes

**Fig. 3:**
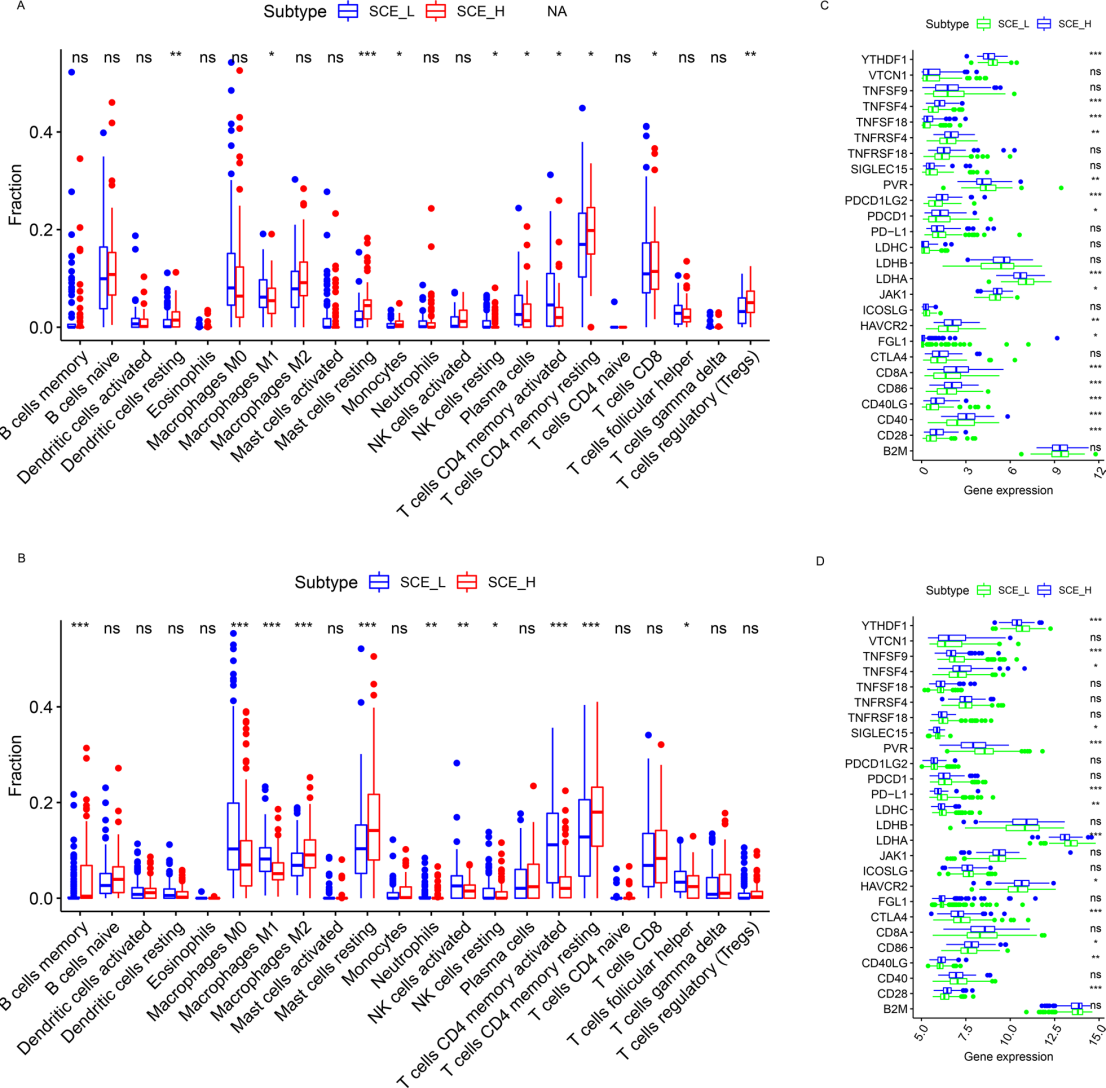
Immune cell infiltration and ICG expression between SCE_L and SCE_H. Comparison of infiltration densities of 22 immune cell types between the **a** TCGA and **b** GSE84437 cohorts. The expression levels of 26 ICGs in SCE_L and SCE_H in the **c** TCGA and **d** GSE84437 cohorts. Statistical significance: *P < 0.05; **P < 0.01; ***P < 0.001

According to the results of BP analysis and GSEA, the TME scores and immune cell fraction of GC stem cell subtypes were investigated. Unanimously, it was found that immune/stromal scores of SCE_H were significantly higher than those of SCE_L (TCGA cohort: Fig. 2d, e; GSE84437 dataset: Fig. 2g, h, all *P* < 0.001), while tumor purity was lower in SCE_L (TCGA cohort: Fig. 2f; GSE84437 dataset: Fig. 2i, all *P* < 0.001). According to the CIBERSORT algorithm (TCGA cohort: Fig. 3a; GSE84437 dataset: Fig. 3b), the infiltration density of M2 macrophages (*P* < 0.05), regulatory T cells (*P* < 0.05), resting mast cells (*P* < 0.05), and memory resting CD4+ T cells (*P* < 0.05), which predicted worse OS [35], increased significantly in SCE_H; while the infiltration density of memory-activated CD4+ T cells (*P* < 0.05), follicular helper T cells (*P* < 0.05), M0 macrophages (*P* < 0.05), and M1 macrophages (*P* < 0.05), which were correlated with better OS [35], decreased significantly in SCE_H. To explain this difference in the infiltration fraction of immune cells between SCE_H and SCE_L, we found that most ICGs were significantly overexpressed in SCE_H (TCGA cohort: Fig. 3c, GSE84437 dataset: Fig. 3d), including *CD28* (*P* < 0.001), *CD40LG* (*P* < 0.01), *CD86* (*P* < 0.05), *HAVCR2* (*P* < 0.05), *TNFSF4* (*P* < 0.05), *PDCD1* (*PD-1*), *CD8A*, *JAK1*, *LDHB*, *PDCD1LG2*, *TNFRSF4*, *TNFRSF18*, and *TNFSF18*; while only a few ICGs were up-regulated in SCE_L (TCGA cohort: Fig. 3c, GSE84437 dataset: Fig. 3d), including *PVR* (*P* < 0.01), *LDHA* (*P* < 0.001), *YTHDF1* (*P* < 0.001), *CD274* (*PD-L1*), and *CTLA4*. The abnormal expression of ICGs could partly explain the differential infiltration of immune cells, and thus, could guide immunotherapeutic strategies based on GC stem cell subtypes.

### Somatic mutation landscape and TMB of GC stem cell subtypes

**Fig. 4:**
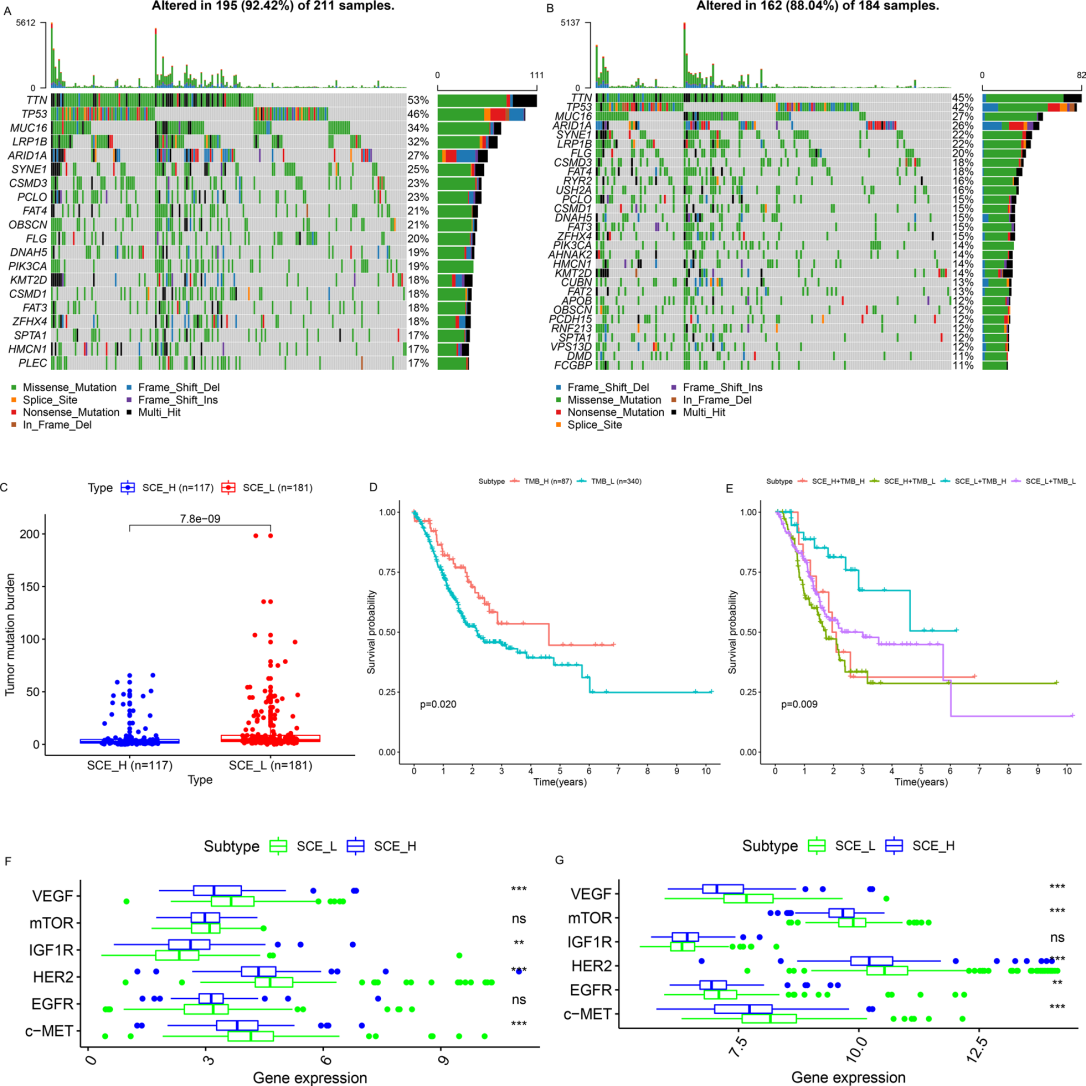
Somatic mutation landscape, TMB, and targeted therapeutic response prediction for GC stem cell subtypes. Somatic mutation landscape of **a** SCE_L and **b** SCE_H. **c** Comparison of TMB between SCE_L and SCE_H. **d** Kaplan–Meier analysis of TMB. **e** Joint survival analysis of GC stem cell subtypes and TMB. The expression levels of six target genes in the **f** TCGA and **g** GSE84437 cohorts

Based on the TCGA cohort, it was found that the mutation frequency of the top 30 genes in SCE_L (Fig. 4a) was much higher than that in SCE_H (Fig. 4b). Moreover, the TMB value of SCE_L was also significantly higher than that of SCE_H (Fig. 4c); higher TMB predicted better OS (Fig. 4d). Further, the joint survival analysis of GC stem cell subtypes and TMB showed that the OS of SCE_L+TMB_H was the best, followed by SCE_L+TMB_L and SCE_H  MB_H, and that of SCE_H+TMB_L was the worst (Fig. 4e).

### Evaluation of targeted therapeutic and chemotherapeutic responses for GC stem cell subtypes

According to differential expression analysis, it was found that five target genes (*HER2*, *EGFR*, *VEGF*, *c-MET*, and *mTOR*) were highly expressed in SCE_L; while only *IGF1R* was significantly up-regulated in SCE_H (TCGA cohort: Fig. 4f; GSE84437 dataset: Fig. 4g). This indicated that patients with the SCE_L subtype may benefit more from targeted therapy.

**Fig. 5:**
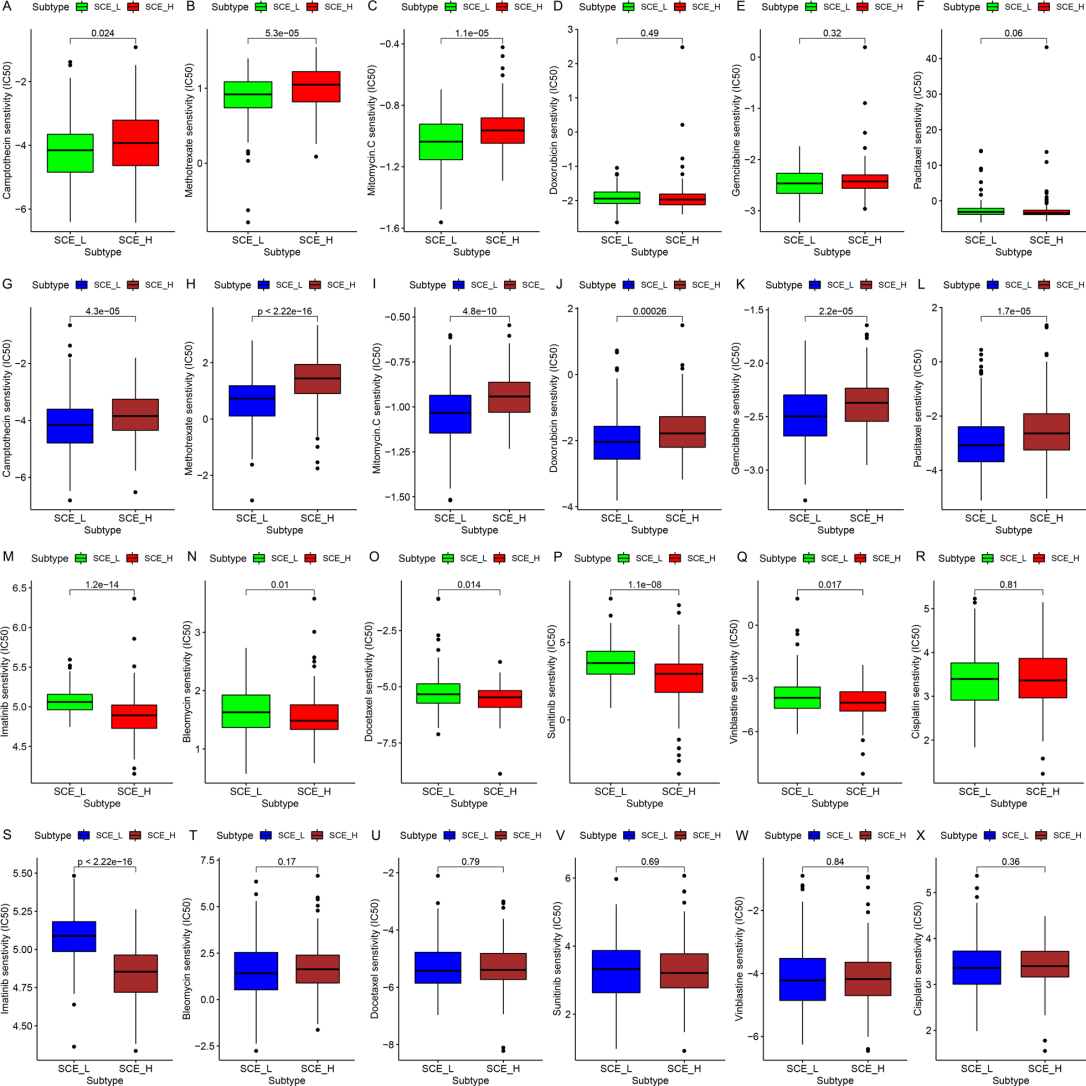
Prediction of chemotherapeutic response for GC stem cell subtypes. Sensitivity of **a** camptothecin, **b** methotrexate, **c** mitomycin C, **d** doxorubicin, **e** gemcitabine, **f** paclitaxel, **m** imatinib, **n** bleomycin, **o** docetaxel, **p** sunitinib, **q** vinblastine, and **r** cisplatin in SCE_L and SCE_H in the TCGA cohort. Sensitivity of **g** camptothecin, **h** methotrexate, **i** mitomycin C, **j** doxorubicin, **k** gemcitabine, **l** paclitaxel, **s** imatinib, **t** blemycin, **u** docetaxel, **v** sunitinib, **w** vinblastine, and **x** cisplatin in SCE_L and SCE_H in the GSE84437 dataset

The half-maximum inhibitory concentration, the IC50 of 12 chemotherapeutic agents were estimated in the samples from the TCGA and GSE84437 datasets. The sensitivity of SCE_L to camptothecin (Fig. 5a, g; all *P* < 0.05), methotrexate (Fig. 5b, h; all *P* < 0.001), mitomycin C (Fig. 5c, i; all *P* < 0.001), doxorubicin (Fig. 5d, j; GSE84437 dataset: *P* < 0.001), gemcitabine (Fig. 5e, k; GSE84437 dataset: *P* < 0.001), and paclitaxel (Fig. 5f, l; GSE84437 dataset: *P* < 0.001) was higher than that SCE_H; the sensitivity of SCE_H to imatinib (Fig. 5m, s; all *P* < 0.001), bleomycin (Fig. 5n, t; TCGA cohort: *P* = 0.01), docetaxel (Fig. 5o, u; TCGA cohort: *P* = 0.014), sunitinib (Fig. 5p, v; TCGA cohort: *P* < 0.001), and vinblastine (Fig. 5q, w; TCGA cohort: *P* = 0.017) was significantly better as compared to SCE_L, and there was no significant difference in the sensitivity of the two stem cell subtypes to cisplatin (Fig. 5r, x). These results provide crucial reference for the chemotherapeutic strategies based on GC stem cell subtypes.

### Potential small molecule drugs based on DEGs in GC stem cell subtypes

Based on the common DEGs (SCE_H/SCE_L) in the TCGA and GSE84437 datasets, we examined 12 small molecule drugs in the CMAP database. These included depudecin, AH-6809, H-89, pivmecillinam, convolamine, azapropazone, benzbromarone, triamcinolone, W-13, cloxacillin, iopromide, and carteolol (Table 2). These drugs could negatively regulate the expression levels of DEGs; thus, alteration of expression levels of the up-regulated/down-regulated genes in SCE_H may help improve the prognoses of SCE_H patients, and the findings may provide a reference for future drug research.

**Table 2:** Potential small molecule drugs for SCE_H patients based on CMAP database

Drugs (inhibitors)	Correlation coefficient	*P* value
Depudecin	− 0.774	0.00217
AH-6809	− 0.705	0.01563
H-89	− 0.685	0.0025
Pivmecillinam	− 0.564	0.02037
Convolamine	− 0.534	0.00064
Azapropazone	− 0.49	0.00437
Benzbromarone	− 0.455	0.01078
Triamcinolone	− 0.455	0.02243
W-13	− 0.451	0.03879
Cloxacillin	− 0.383	0.00901
Iopromide	− 0.382	0.04279
Carteolol	− 0.316	0.04062

### Generation, evaluation, and validation of prognostic risk scoring model

**Fig. 6:**
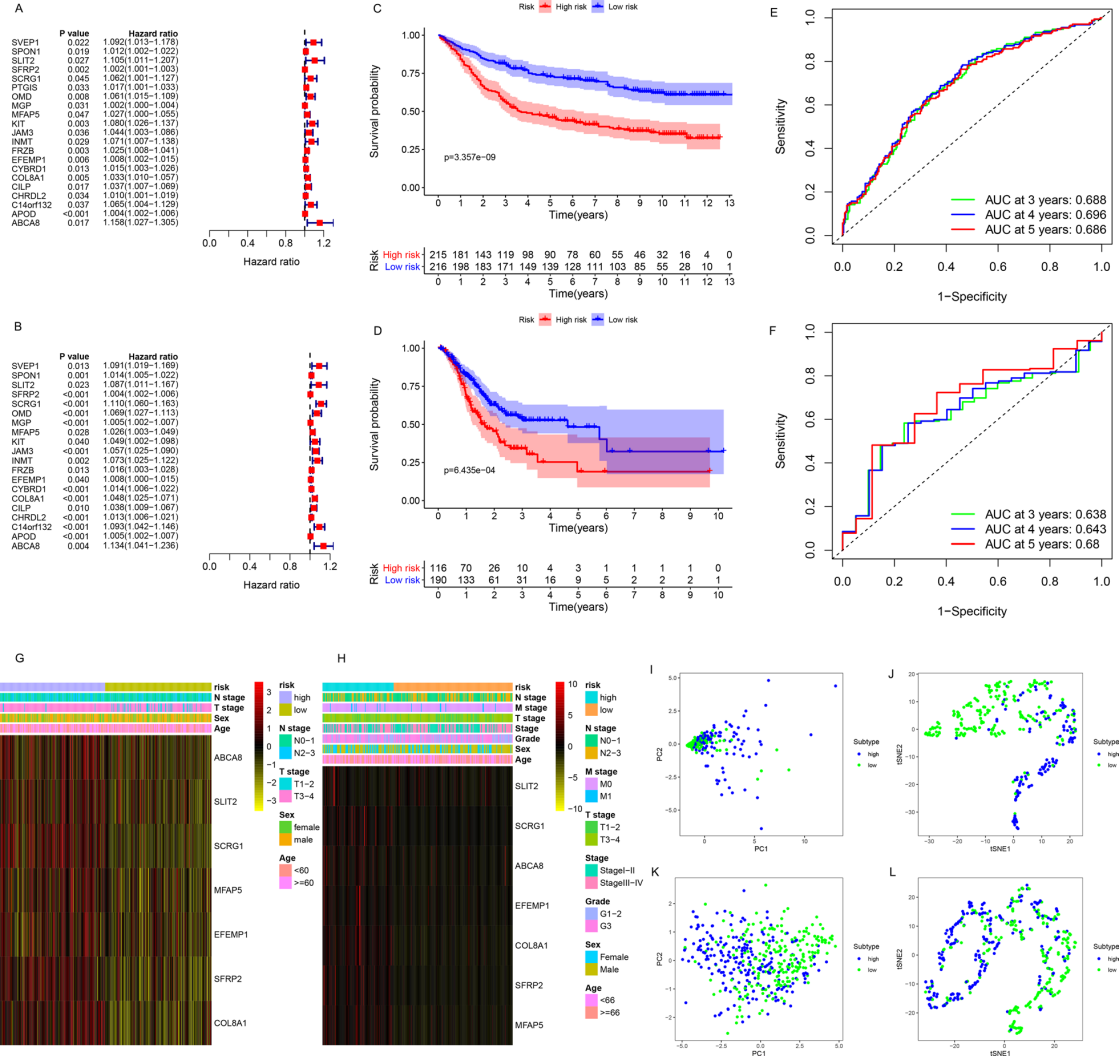
Construction and evaluation of a prognostic risk scoring model. Univariate analysis of the **a** training and **b** validation sets. Kaplan–Meier analysis of the **c** training and **d** validation sets. ROC curves of the **e** training and **f** validation sets for predicting 3-, 4-, and 5-year OS. The expression of seven genes and the proportion of clinicopathological features in the high- and low-risk groups were visualized in both **g** training and **h** validation sets. Clustering of patients belonging to high- and low-risk groups in the **i**, **j** training and **k**, **l** validation sets based on PCA and tSNE algorithm

**Fig. 7:**
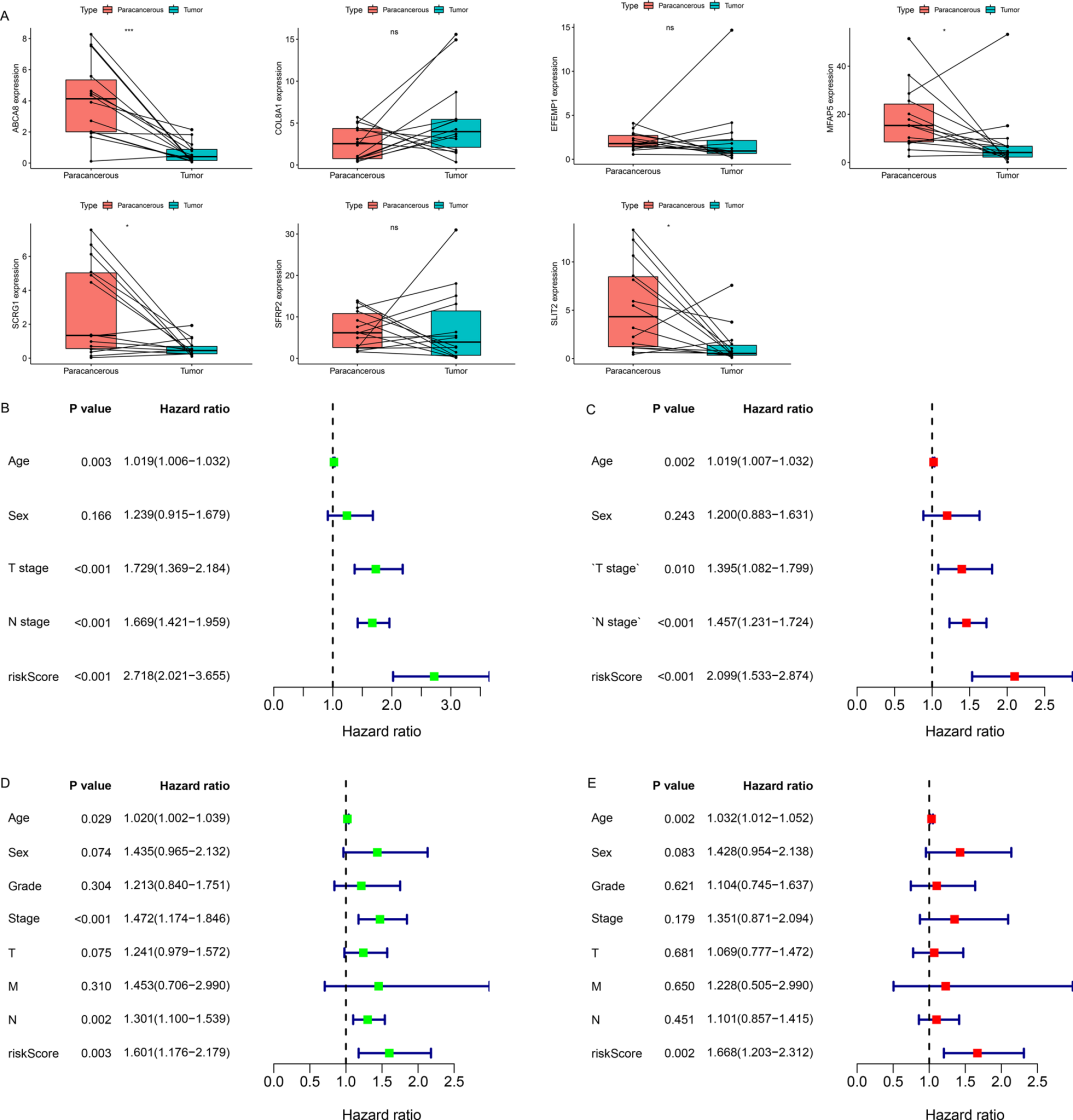
qPCR analysis for seven genes and independent test of risk scoring model. **a** The qPCR analysis of expression levels of seven genes in GC samples (*n* = 14) and paired para-cancerous samples (*n* = 14). **b** Univariate and **c** multivariate analysis of the training set. **d** Univariate and **e** multivariate analysis of the validation set. Statistical significance: *P < 0.05; **P < 0.01; ***P < 0.001

A total of 21 co-prognostic genes were obtained after the intersection of the prognostic DEGs between the training (Fig. 6a) and the validation sets (Fig. 6b). Subsequently, these were used for the multivariate Cox regression analysis, and a seven-gene-based RS model was generated to predict patient OS. The RS of each sample was calculated based on the expression of the seven genes and their relative coefficients as follows: RS = (− 0.295886 × expression of *SLIT2*) + (0.0046356 × expression of *SFRP2*) + (0.1338614 × expression of *SCRG1*) + (− 0.043459 × expression of *MFAP5*) + (− 0.014278 × expression of *EFEMP1*) + (0.0872369 × expression of *COL8A1*) + (0.1638184 × expression of *ABCA8*). Kaplan–Meier analysis demonstrated that both in training (Fig. 6c, P = 3.357e − 09) and validation sets (Fig. 6d, P = 6.435e − 04), the OS of the low-risk group was better than that of the high-risk group. Additionally, the expression of the seven genes in the high-risk group was higher than that in the low-risk group (Fig. 6g); patients in the high-risk group had worse clinical results and pathological stages (Fig. 6g). Similar results were obtained in the validation set (Fig. 6h). The areas under the ROC curves for predicting 3-, 4- and 5-year OS were 0.688, 0.696, and 0.686, respectively, in the training set (Fig. 6e), and 0.638, 0.643, and 0.680, respectively, in the validation set (Fig. 6f). Patients in high- and low-risk groups could also be distinguished based on PCA (training set: Fig. 6i; validation set: Fig. 6k) and tSNE algorithm (training set: Fig. 6j; validation set: Fig. 6l). These results indicated that the RS model had high accuracy of prediction. In addition, the qPCR analysis estimated the expression levels of the seven genes in samples; among them, expression of ABCA8 (*P* < 0.001), MFAP5 (*P* < 0.05), SCRG1 (*P* < 0.05), and SLIT2 (*P* < 0.05) were significantly lower in the GC samples (Fig. 7a).

Univariate and multivariate analyses were performed to eliminate the interfering factors. In the training set, univariate analysis showed that age (*P* = 0.003), T stage (*P* < 0.001), N stage (*P* < 0.001), and RS (*P* < 0.001) were the prognostic factors; the hazard ratio (HR) of RS was the highest (Fig. 7b). Further, multivariate analysis showed that RS had a strong independent prediction ability (Fig. 7c: *P* < 0.001, HR = 2.099). Similar results were obtained in the validation set (Fig. 7d, e).

### Biological significance of RS

**Fig. 8:**
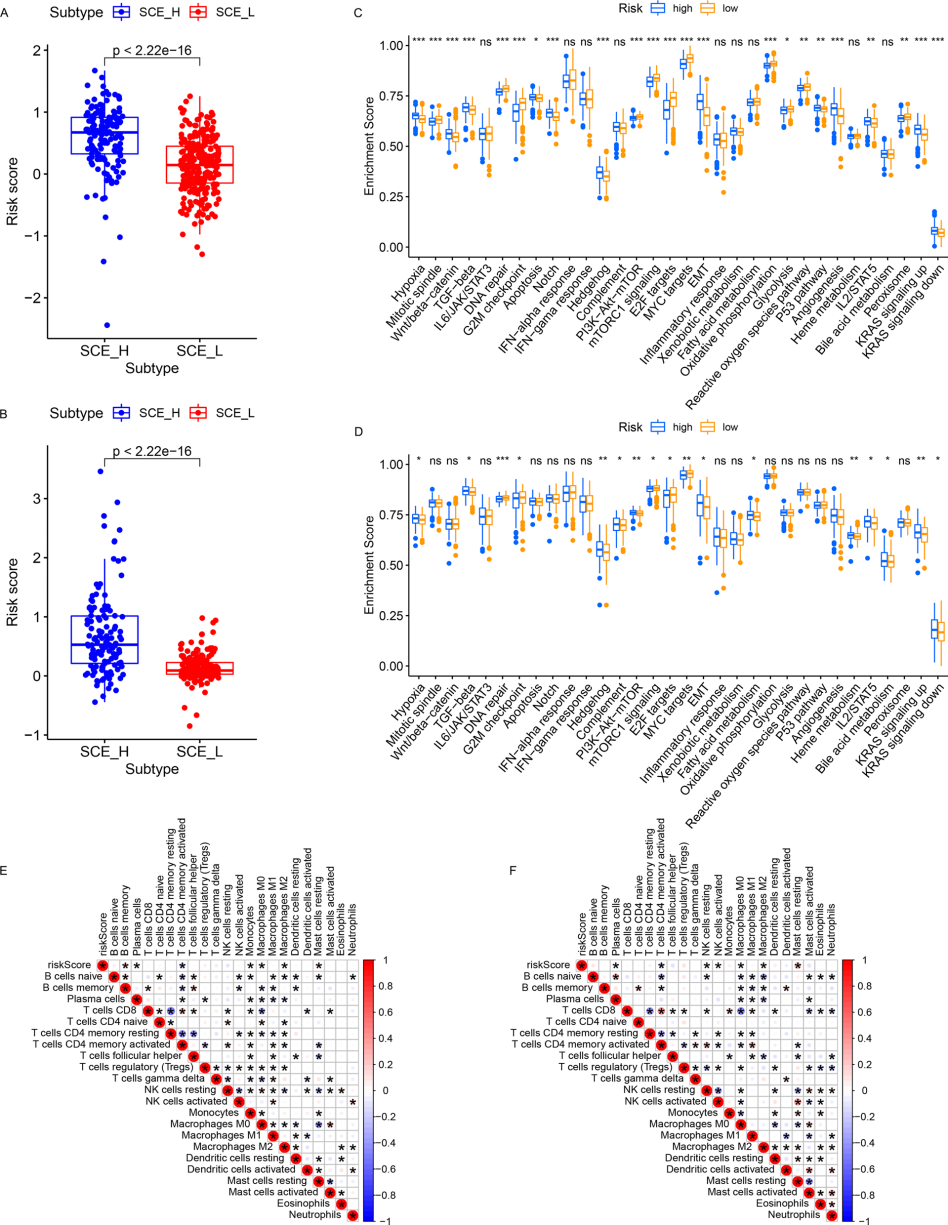
The biological significance of RS. **a**, **b** Differences in RS between the two stem cell subtypes in **a** the training and **b** validation sets (all *P* < 2.22e − 16). **c**, **d** Two stem cell subtypes were distinguished by well-defined biological signatures curated from MSigDB database in **c** the training and **d** validation sets. Statistical significance: *P < 0.05; **P < 0.01; ***P < 0.001. **e**, **f** The correlation between RS and TME infiltration cells using Spearman’s analysis in **e** the training and **f** validation sets. Negative correlation: blue; positive correlation: red. *P* < 0.05

Differential analysis showed that both in training (Fig. 8a, P < 2.22e − 16) and validation sets (Fig. 8b, P < 2.22e − 16), the RS of SCE_H was significantly higher than that of SCE_L. This suggested that the poor prognoses in the high-risk group were closely related to CSCs. Moreover, as compared with the low-risk group, the high-risk group showed stromal activation (e.g., hypoxia, EMT, and angiogenesis), classical oncogenic pathway activation (e.g., Wnt/β-catenin pathway, TGF-β signaling pathway, Notch signaling pathway, and p53 pathways), and post-mutation repair arrest (e.g., DNA repair and G2M checkpoint recovery) (Fig. 8c, d). Correlation analysis further indicated that RS was negatively correlated with the infiltration fractions of plasma cells (*P* < 0.05), activated memory CD4+ T cells (*P* < 0.05) and M0 macrophages (*P* < 0.05), while positively associated with the infiltration fractions of monocytes (*P* < 0.05), M2 macrophages (*P* < 0.05), and resting mast cells (*P* < 0.05) (Fig. 8e, f).

## Discussion

GC is a primary malignant tumor with strong heterogeneity, and can seriously endanger human health [36]. The importance of GC heterogeneity for patient therapeutic response and the prognostic assessment for tumor site [37], tissue type [38], pathological type, early or advanced stage [39], stage of treatment [40], and primary or metastatic focus [41] have been recognized. With the rapid development of molecular biology and molecular diagnostic technology, the intratumoral heterogeneity of GC has been evaluated at the molecular level. For example, molecular phenotypic identification (e.g., HER2 and VEGF) optimizes diagnoses and treatment strategies and promotes the development of precision medicine or targeted therapy [29–33]; molecular typing (e.g., Lei typing, TCGA typing, and ACRG typing) contributes to stratified diagnosis and treatment [17–19]. Since the establishment of GC molecular typing is in its infancy, and different molecular typing methods do not have a simple correlation between each other, this study further evaluated the GC heterogeneity from the perspective of the genome of human stem cells. The identification of GC stem cell subtypes could accurately predict the patient’s clinical outcomes, TME status, immune cell infiltration, ICG expression, somatic mutation landscape, potential targeted therapy, and chemotherapeutic response. A CSC typing-related RS model was prudently generated and validated by cross-platform datasets and different algorithms.

CSCs refer to a small number of tumor cells with self-renewal and strong reproductive ability. They can differentiate into a large number of new tumor cells, and have a vital relationship with the occurrence, development, metastasis, and prognosis of tumors [42]. To date, numerous studies have revealed the effect of CSCs in occurrence, recurrence, distant metastasis, and drug resistance of GC [13, 14]. The prognosis of SCE_H, including survival and clinicopathological outcomes, was worse in this study, and the findings were consistent with previous reports. Investigations of the underlying mechanisms showed that the classical oncogenic pathways, immune escape, and regulation of stem cell division were highly activated in SCE_H; while ferroptosis, immune response, and post-mutation repair pathways were closely associated with SCE_L. Further analysis showed that SCE_H had higher immune infiltration and stromal components, with lower tumor purity; the difference observed in immune infiltration was in contradiction with the previous view that higher immune infiltration was strongly correlated with better prognosis. To further explain this phenomenon, CIBERSORT analysis showed that multiple cytotoxic lymphocytes were significantly reduced in SCE_H, while the fraction of M2 macrophages and regulatory T cells involved in the immune escape was higher. Previous studies have found that M2 macrophages with immunosuppressive properties can promote tumor immune escape by upregulation of non-classical MHC class I molecules (e.g., HLA-E and HLA-G), inhibitory ligands for T cells, apoptosis receptors (e.g., PD-L1, TRAIL, and B7-H4), and SIRP-alpha [43, 44]. Moreover, M2 macrophages can accelerate the malignant progression of tumors by promoting angiogenesis and tumor cell migration [43, 44]. Regulatory T cells are a unique subset of CD4+ T cells with immunosuppressive properties; these are essential for maintaining immune homeostasis, self-tolerance, limiting excessive inflammation, and preventing autoimmunity [45, 46]. In cancers, regulatory T cells can inhibit anti-tumor immune response, and thus, are considered to be the major obstacle of tumor immunotherapy; these are recruited into TME through chemokines secreted by tumor cells and M2 macrophages [45, 46]. Therefore, the identification of stem cell subtypes could accurately reveal the TME cell-infiltrating characteristics of patients with prognostic differences. Moreover, the findings also showed that a large number of ICGs were highly expressed in SCE_H. Abundant reports have shown that the up-regulation of ICGs on the surface of tumor cells is a key factor of tumor immune escape [28, 47, 48]. ICGs can suppress the proliferation and differentiation of T lymphocytes, promote the differentiation of Tregs, and induce the secretion of cytokines, thereby suppressing the immune response [49]. The elevated expression of ICGs indicated that patients with the SCE_H subtype may respond better to immunotherapy.

Cancer is a disease of abnormal cell proliferation caused by somatic gene mutations, which mainly occur in the process of repair of DNA damage, DNA replication, cell division, and nucleic acid metabolism [50, 51]. Under the influence of external physical or chemical mutagenesis factors, the number of somatic gene mutations increases further [50, 51]. Therefore, the range, type, and frequency of gene mutations, collectively known as TMB, can be quite different due to the differences in tumor types, living environments, and genetic characteristics [52]. TMB can cause changes in protein sequences; these abnormally expressed proteins can act as new antigens which can bind to type I or type II major histocompatibility complex and be recognized by the immune system when presented on the cell surface, thereby activating T lymphocytes to produce immune response [50, 51]. Therefore, the immunogenicity of tumors is closely related to TMB; higher TMB tends to induce local immune recognition and improves patient survival and clinicopathological outcomes. Our study showed that patients with SCE_L subtype had a higher frequency of genetic mutations and TMB, which implied that SCE_L probably had more expression of neoantigens recruiting lymphocyte infiltration. Therefore, the identification of stem cell subtypes could reasonably explain the characteristics of TME cell-infiltration from the perspective of somatic mutations.

The biological characteristics of CSCs (e.g., cell cycle arrest, DNA damage tolerance and repair, drug efflux, and epithelial-mesenchymal transition) [6, 7] and TME (e.g., hypoxia, tumor-associated fibroblasts, and chronic inflammation) [53–56] jointly sustain cancer stemness. This hinders the chemotherapeutic stimulation on CSCs and increases the difficulty of tumor therapy. For instance, Haraguchi et al. [57] show that GD15 can increase the cell proportion of hepatic CSCs in G0/G1 phase by AKT/GSK-3β/β-catenin signaling pathway, enhance the ability of hepatic CSCs to form a ball, and increase its resistance to chemotherapy. Sun et al. [54] show that activation of the PI3K/ATK signaling pathway upregulates HIF-1α in a hypoxic environment, and further, enhances the chemotherapeutic resistance of CSCs. Currently, GC is mainly treated by surgery and chemotherapy. Hence, understanding the chemosensitivity of GC stem cell subtypes is of great clinical relevance. Our study showed that SCE_L was more sensitive to camptothecin, methotrexate, mitomycin C, doxorubicin, gemcitabine, and paclitaxel; while SCE_H was more sensitive to imatinib, bleomycin, docetaxel, sunitinib, and vinblastine. Therefore, the identification of stem cell subtypes provides a crucial reference for patients undergoing chemotherapy. In addition, small molecule drug screening provided new insights for exploring the mechanisms of drug resistance of CSCs. This may help in developing new chemical drugs and thus improve the curative effect of GC.

Recent studies have focused on the establishment of prognostic RS models based on protein-coding genes [35], non-coding genes (e.g., lncRNA, miRA, and circRNA) [58], and CpG island methylation sites [59] to evaluate survival outcomes in GC patients. As the generation of the model is in the preliminary research stage, there is still a lack of a widely accepted model for clinical applicability. Given that the gene-based RS model may be useful for predicting patient OS, an RS model based on prognostic DEGs between GC stem cell subtypes was constructed; it had high accuracy and prediction efficiency. Further analysis showed that RS was highly correlated with the two stem cell subtypes and the differences between high- and low-risk groups were similar to those in SCE_H and SCE_L for biological pathways and TME cell-infiltrating characteristics. Therefore, the RS model is a genetic model associated with CSC typing, which may fundamentally elucidate tumor heterogeneity. It deserves further clinical prospective studies.

However, the study has certain limitations. First, although bioinformatic analysis-based GC stem cell subtypes have been validated by multiple datasets and algorithms, robust experimental studies are necessary to gain more insight into the underlying mechanisms of the GC stem cell subtypes. Therefore, we are collecting clinical samples to verify these results; however, this will be time-consuming. Second, an independent external dataset and various methods were utilized to confirm the RS modeling algorithm, which was optimal for the current study, however, the RS model was constructed and validated based on retrospective data from publicly available open databases. Thus, large-scale prospective clinical research is required to evaluate its effectiveness and practicability.

## Conclusion

The identification of GC stem cell subtypes could accurately predict patient clinical outcomes, TME cell-infiltrating characteristics, somatic mutation landscape, and potential responses to immunotherapy, targeted therapy, and chemotherapy. The CSC typing-related RS model provided an intuitive and accurate method for predicting patient OS. These results revealed the complex oncogenic mechanisms underlying GC and proposed a promising direction for the diagnoses and treatment strategies for GC.

## Supplementary Information

**Additional file 1. Table S1**: The clinical and demographic features of gastric cancer patients from Renmin Hospital of Wuhan University.

**Additional file 2. Fig. S1**: Identification of GC stem cell subtypes based on the GSE84437 dataset. (A–C) Two stable stem cell subtypes were identified using consensus clustering analysis according to the K-means algorithm and CDF curve. (D) GC subtypes were classified as SCE_L and SCE_H based on 26 stem cell gene sets. Clustering of patients belonging to SCE_L and SCE_H in the TCGA cohort based on PCA and tSNE algorithm. Clustering of patients belonging to SCE_L and SCE_H in the GSE84437 dataset based on (E) PCA and (F) tSNE algorithm. (G) The expression of 26 stem cell gene sets and the proportion of clinicopathological features in SCE_L and SCE_H. (H) Kaplan-Meier analysis of GC stem cell subtypes. Statistical significance: *P < 0.05; **P < 0.01; ***P < 0.001.

## Data Availability

Twenty-six stem cell gene sets were obtained from the StemChecker portal (http://stemchecker.sysbiolab.eu/). The bulk RNA-seq data of GC samples were accessed from the TCGA (http://cancergenome.nih.gov/) and GEO databases (GSE84437, https://www.ncbi.nlm.nih.gov/geo/).

## Abbreviations

GC: Gastric cancer
RS: Risk score
DEGs: Differentially expressed genes
OS: Overall survival
SCE_L: Low stem cell enrichment
SCE_H: High stem cell enrichment
CSC: Cancer stem cell
TME: Tumor microenvironment
ICGs: Immune checkpoint genes
TCGA: The Cancer Genome Atlas
ACRG: The Asian Cancer Research Group
ssGSEA: Single-sample gene set enrichment analysis
CDF: Cumulative distribution function
PCA: Principal component analysis
tSNE: T-Distributed stochastic neighbor embedding
FDR: False discovery rate
BP: Biological process
GSEA: Gene set enrichment analysis
FC: Fold change
TMB: Tumor mutation burden
GDSC: Genomics of Drug Sensitivity in Cancer
CMAP: Connectivity map
ROC: Receiver operating characteristic
qPCR: Quantitative PCR
HR: Hazard ratio
MSI: Microsatellite instability
EBV: Epstein–Barr virus
