# Supplementary figures and images for: Identification of stem cell-related subtypes and risk scoring for gastric cancer based on stem genomic profiling
Source: Stem Cell Res Ther. 2021 Oct 30;12:563. doi: 10.1186/s13287-021-02633-x (PMC8557621; doi:10.1186/s13287-021-02633-x)

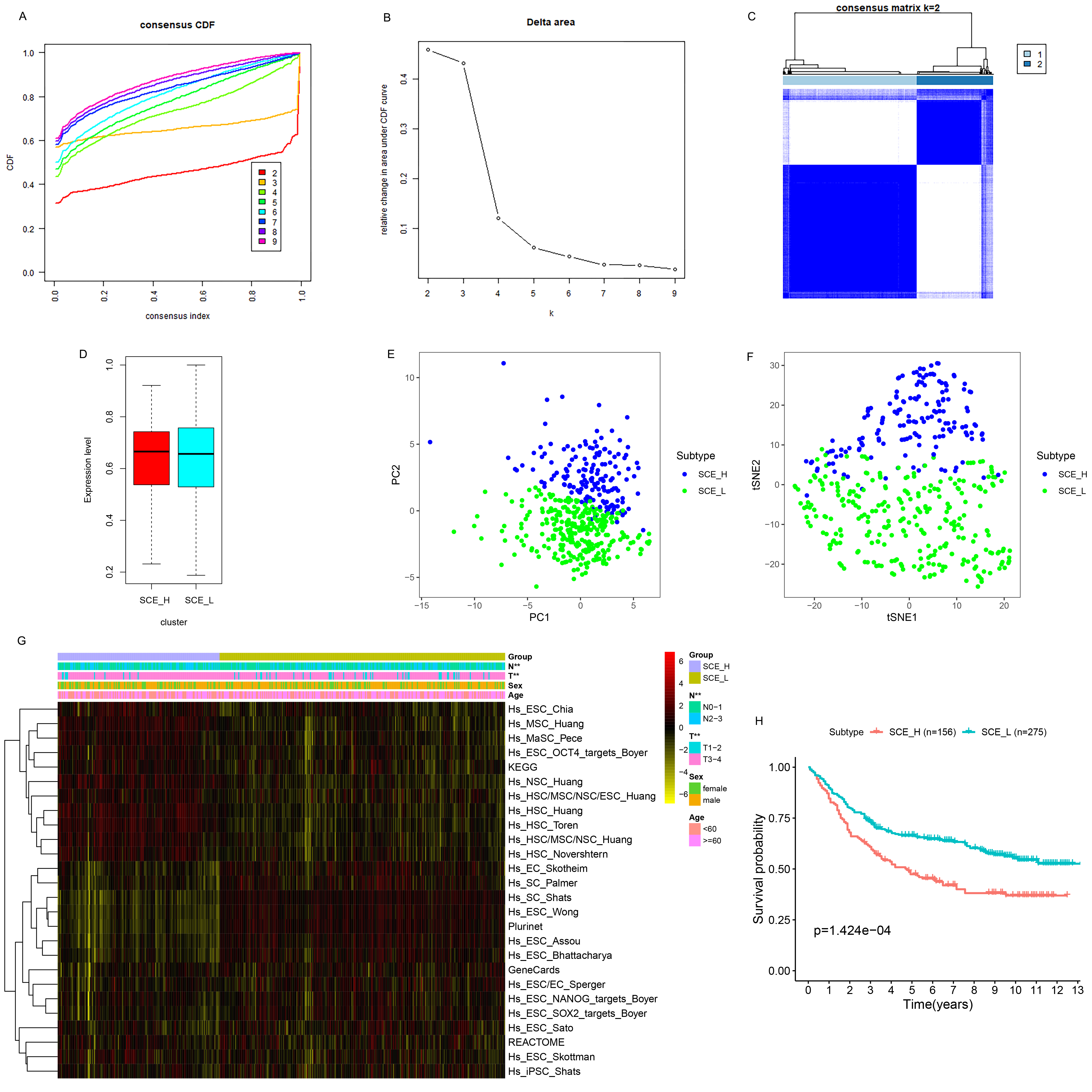

Supplement: Supplementary file 2 — Additional file 2. Fig. S1: Identification of GC stem cell subtypes based on the GSE84437 dataset. (A–C) Two stable stem cell subtypes were identified using consensus clustering analysis according to the K-means algorithm and CDF curve. (D) GC subtypes were classified as SCE_L and SCE_H based on 26 stem cell gene sets. Clustering of patients belonging to SCE_L and SCE_H in the TCGA cohort based on PCA and tSNE algorithm. Clustering of patients belonging to SCE_L and SCE_H in the GSE84437 dataset based on (E) PCA and (F) tSNE algorithm. (G) The expression of 26 stem cell gene sets and the proportion of clinicopathological features in SCE_L and SCE_H. (H) Kaplan-Meier analysis of GC stem cell subtypes. Statistical significance: *P < 0.05; **P < 0.01; ***P < 0.001. [file 13287_2021_2633_MOESM2_ESM.tif]
